# The coronavirus crisis as tipping point: communicating the environment in a time of pandemic

**DOI:** 10.1177/1329878X20950030

**Published:** 2020-11

**Authors:** Gabi Mocatta, Erin Hawley

**Affiliations:** Deakin University, Australia

**Keywords:** climate change, COVID-19, environmental communication, media discourse, tipping point

## Abstract

This essay examines media and environment during the pandemic through the conceptual lens of environmental communication. We take the pulse of environmental communication under COVID-19, noting that while the quantity of media coverage on key environmental issues has fallen during the blanket coverage of the pandemic, COVID-19 has acted on multiple levels as a moment of discursive change in environmental communication. We contend that mediatised discourse on the environment during the pandemic has offered new insights, and an opportunity for a reset in environmental understandings, including a new consciousness of global connectedness in environmental responsibility, and an opportunity to improve publics’ environmental literacy. *** This essay is intended for the extraordinary issue, on Coronavirus, Crisis and Communication ***

Times of crisis can be moments of fear, in which the future becomes threatening and unknown. Moments of crisis can also be points of inflection: instructive opportunities to rethink old ways of behaving, to pause, re-evaluate, and choose new paths. In this essay, we conceptualise the coronavirus pandemic as such a point of inflection in mediatised discourse on the environment. We briefly examine here a range of mainly Australian media coverage of environmental issues during the COVID-19 pandemic, and we identify some of the key distinguishing framings of media reporting on the environment during this time.

We examine media and environment during the pandemic through the conceptual lens of environmental communication, which like conservation biology, regards itself a ‘crisis discipline’ (Cox, 2007: 4), being fundamentally involved with the revealing and addressing of environmental harms. Environmental communication, as a field of study and practice, invites dialogue about threats to nature and considers as its key normative tenet an obligation to enhance ‘the ability of society to respond appropriately to environmental signals relevant to the wellbeing of both human civilization and natural biological systems’ (Cox, 2007: 4).

In this essay, we take the pulse of environmental communication under COVID-19. We note that while the quantity of media coverage on key environmental issues, such as climate change, may have fallen during the blanket coverage of the pandemic itself, COVID-19 has acted on multiple levels as a pivotal moment of discursive change in environmental communication. We contend that mediatised discourse on the environment during the pandemic has offered new insights, and an opportunity for a reset in environmental understandings, including a new consciousness of global connectedness in environmental responsibility, and an opportunity to improve publics’ environmental literacy.

## Media and environment through the pandemic

Coronavirus has been a media spectacle. The vast, agenda setting media coverage of the pandemic has lasted for months, occupying more mediatised space and generating more social concern and panic (Wahl-Jorgensen, 2020) than perhaps any global event since the World Wars. This unwavering focus on the pandemic has eclipsed coverage of other issues. Early analysis shows that media attention to climate change dropped dramatically during the early months of the pandemic. A global monitoring project that tracks media coverage of climate change found reporting on this issue down 59% from January to April 2020 (Nacu-Schmidt et al., 2020). In Australia specifically, reporting on climate change fell to less than 1% of media content in March and April 2020, while 80% of articles mentioned coronavirus by the end of March 2020 (Hannam, 2020). In the environment coverage that persisted, however, there seem to have been some qualitative changes, and new, consistent framings have emerged.

One prominent frame in mediatised environmental discourse has been attribution of the pandemic to human incursions into nature, and a realisation that we are not separate from nature, but dependent on it. ‘Nature is sending us a message’, was how several Australian media framed this notion. *The Guardian* quoted the UN’s environment chief, Inger Anderson as saying: ‘We are intimately interconnected with nature, whether we like it or not. If we don’t take care of nature, we can’t take care of ourselves’ (Carrington, 2020). The *ABC* similarly reported that ‘global disregard for nature’ (Diprose and Neal, 2020) had brought about the pandemic, while the *Sydney Morning Herald* wrote of the ‘perfect storm’ for pandemics created by the global illegal wildlife trade (Groch, 2020). ‘We did it to ourselves’ *The Guardian* reported a prominent US biologist as saying. ‘To stop future pandemics’, it wrote ‘we need more respect for the natural world’ (Weston, 2020).

Media also reported on the environment under coronavirus by emphasising the beneficial effects of global lockdown on air pollution, and the reclaiming of human spaces by animals. This was a global theme, reflected in images of dramatically clean skies and waters in usually crowded, polluted cities, and wild animals seen in urban settings. The *BBC* posted a video, for example, of a kangaroo hopping through downtown Adelaide.

Media discourse additionally coalesced around a frame of ‘listening to science’. As politicians deferred to epidemiologists, payed utmost attention to their daily reports of R numbers, and acted decisively on their disease transmission modelling, some in the media began to link this reliance on science with long-standing government dismissal of climate science. ‘Australia listened to the experts on coronavirus. It’s time we heard them on climate change’ wrote *Guardian* editor, Lenore Taylor, for example. The pandemic showed that ‘good decisions are made’ when the government considers ‘the evidence and the best available expert advice’ she wrote, showing that ‘a different kind of politics’ was possible (Taylor, 2020).

Importantly, the pandemic lockdown was also discussed in terms of its capacity to both temporarily and, perhaps permanently, reduce greenhouse gas emissions and stimulate change. The *SBS* described ‘the largest-ever reduction of energy demand and CO2 emissions’. While in the context of deaths and economic trauma this was ‘nothing to celebrate’, it could ‘point the way to structural change’, making the crisis ‘a genuine turning point for world energy markets’ the *SBS* reported (AFP/SBS, 2020). Once the pandemic seemingly reached its (first) peak in Australia, and the initial ‘curve’ had been flattened, there were calls to flatten the (infinitely more threatening) ‘climate change curve’ with a renewables-led economic recovery. That the coronavirus could act as a point of inflection in the fight against climate change was reflected substantially in media discourse. An article in *The Conversation*, for example, called the pandemic a ‘sliding doors moment’ and a ‘fork in the road’ at which we could ‘change Earth’s trajectory’ (Canadell et al., 2020).

While mainstream media turned at least some of its attention to communicating environmental messages during the early days of the pandemic, social media became a site for the spread of human-focused health misinformation and conspiracy theories. In social media discourse, there were claims of miracle cures, and attributions of the virus’ origins variously to a bio-weapon, 5G networks, and the world-dominating plans of vaccine advocates. Australian mainstream media debunked some of these false claims (Bogle, 2020). Several stories that spread globally on social networks did relate to the return of animals while humans were in lockdown – one, for example, purporting to show dolphins swimming in Venice’s canals, a claim since debunked (Daly, 2020). In explanation of such social media content, US psychology Professor Sue Clayton has commented that ‘people really want to believe in the power of nature to recover. People hope that, no matter what we’ve done, nature is powerful enough to rise above it’ (quoted in Daly, 2020). Such ‘fake news’ social media posts are harmful precisely because they spread false hope. However, it might also be argued that despite their untruths, such viral fakes indicate that the new appreciation for nature seen in mainstream media during the early months of the pandemic spread to social media also.

One aspect of environmental communication in the midst of the pandemic was notable for its relative *absence*. Without the ability to hold physical protests under lockdown, environmental activism became much less visible in mainstream media. Climate advocacy groups such as Extinction Rebellion have built their communication strategies on the physical act of flooding public space with visual and verbal messages, signalling a sense of emergency and crisis. Isolation across the globe has shifted action by groups like Extinction Rebellion and Fridays For Future into a fully online space. Although this has enabled grassroots environmental communication to continue, it has diminished these groups’ ability to seize and hold the gaze of the mainstream news audience. Extinction Rebellion was reported in Australian media to be planning ‘digital disruption’ under lockdown. ‘We’re still watching the government and we’ll still hold them to account’, the *Sydney Morning Herald* reported the group’s spokesperson as saying (Fitzsimmons, 2020).

## Writing environmental concerns into ‘the new normal’

In many ways, environmental communication during the pandemic highlights the importance and urgency of environmental literacy, by demonstrating the need for a better understanding of the human/nature relationship and the consequences of human incursions into nature. Environmental literacy incorporates the knowledge and competencies that empower individuals and communities to act, live, and encourage policy action in ways that enable environmental sustainability and reduce planetary harm. Media commentary linking COVID-19 to problems inherent in the human/nature relationship ensures that environmental sustainability as ‘a vision of achieving human and eco-system well-being *together*’ (Washington, 2015: 2) has quickly moved from the margins to the centre of media discourse: where it remains, as we begin to consider our emergence into a post-pandemic world. Media frequently noted during the pandemic that we are ‘all in this together’ (7News, 2020). Such statements highlight global interconnectedness and responsibility in terms of the pandemic, and they likewise underscore the nature of global *environmental* impacts and responsibilities.

In addition, while the pandemic may have largely shifted media (and public) attention onto *human* (rather than planetary) suffering and wellbeing, and onto the needs of present rather than future generations, there are close parallels to be drawn between the two crises. In research commissioned by European Climate Foundation, the charity group Climate Outreach reported that ‘Covid-19 can feel like a sped up analogy for climate change’ because ‘Both are major health challenges, presenting a global threat to wellbeing in which the vulnerable are hit first and hardest, and personal and local action play a crucial role’ (Webster et al., 2020: 7).

Environmental communicators have seized upon these links between the two crises in order to maintain the momentum of concern for planetary health. For example, in a call for continued ‘rebellion’ against government and industry during the pandemic, Clare Farrell (2020), co-founder of Extinction Rebellion, stated ‘Health, environment, inequality, planetary stability – it’s all connected, and as a global family, so are we’. Meanwhile Christiana Figueres, former head of the UN Climate Change Convention and architect of the Paris Agreement, has said that ‘The Covid-19 pandemic has unleashed humanity’s instinct to transform itself in the face of a universal threat and it can help us do the same to create a livable planet for future generations’ (Carbon Brief, 2020). Jennifer Morgan, Executive Director of Greenpeace, has described the pandemic as a chance for ‘reset’ and called for ‘a just and climate-safe world, because environmental health and our own well-being are dependent on each other’ (Greenpeace International, 2020).

It has been argued that humans have a limited capacity to be worried about multiple problems at the same time: a ‘finite pool of worry’ (Weber, 2006). Yet interestingly, while there has been a drop in news coverage of climate and environmental crisis during the pandemic, public concern about climate change has not necessarily declined (Schwartz, 2020). A poll conducted in April 2020 found widespread global support for ‘government actions to prioritise climate change in the economic recovery after COVID-19’ (IPSOS, 2020). This suggests that far from experiencing ‘worry fatigue’, and despite the trauma and suffering caused by the pandemic, audiences and publics may well be in a state of hyper-awareness, openness to emergency, and readiness to respond. The upswelling of public support for the Black Lives Matter movement in the wake of George Floyd’s death, and while still in the pandemic’s grip, demonstrates that one ‘worry’ does not simply displace others, especially when these combined ‘worries’ shine a light on the same deeply ingrained problems of injustice, inequality, and the uneven distribution of suffering – including suffering wrought by environmental harms.

Media reporting on climate and the environmental crisis may have contracted in quantity during the pandemic, as individuals, communities, and governments dealt with more immediately pressing concerns relating to health, human suffering and economic woes. While acknowledging this drop in quantity, our paper has explored the shifts and changes in the *quality* of environmental communication during the COVID-19 crisis. Arguably, we can conceive of the pandemic not just as a distraction from the environmental crisis but as a tipping point in the way that we understand our interactions with nature – one that offers a unique opportunity to write environmental sustainability into our ‘new normal’.
